# From smoke to symbiosis: dissecting KAI2 signaling in rice using the specific receptor agonist, dMGer

**DOI:** 10.1093/plphys/kiag389

**Published:** 2026-06-17

**Authors:** James M Bradley

**Affiliations:** Assistant Features Editor, Plant Physiology, American Society of Plant Biologists, Rockville, MD 20855-2768, United States; Department of Cell & Systems Biology, University of Toronto, 25 Willcocks Street, Toronto, ON M5S 3B2, Canada

Forest fires release smoke-derived chemicals called karrikins (KARs) that trigger germination of “fire-following” plants, ensuring germination coincides with low competition and plentiful resources. Genetic analyses in Arabidopsis revealed an α/β-hydrolase receptor, KAI2 (D14L in rice), is required for KAR responses. KAI2 shares structural similarity with its paralog, D14, which perceives the plant hormone strigolactone (SL) and modulates aspects of plant development, such as shoot branching (Aquino et al 2020). Both KAI2 and D14 require the same F-box protein, MAX2 (D3 in rice), to target downstream regulators of the SMAX1-LIKE (SMXL) gene family for proteasomal turnover (Fig. 1a) (Nelson et al 2011; Conn and Nelson 2016).

**Figure 1:**
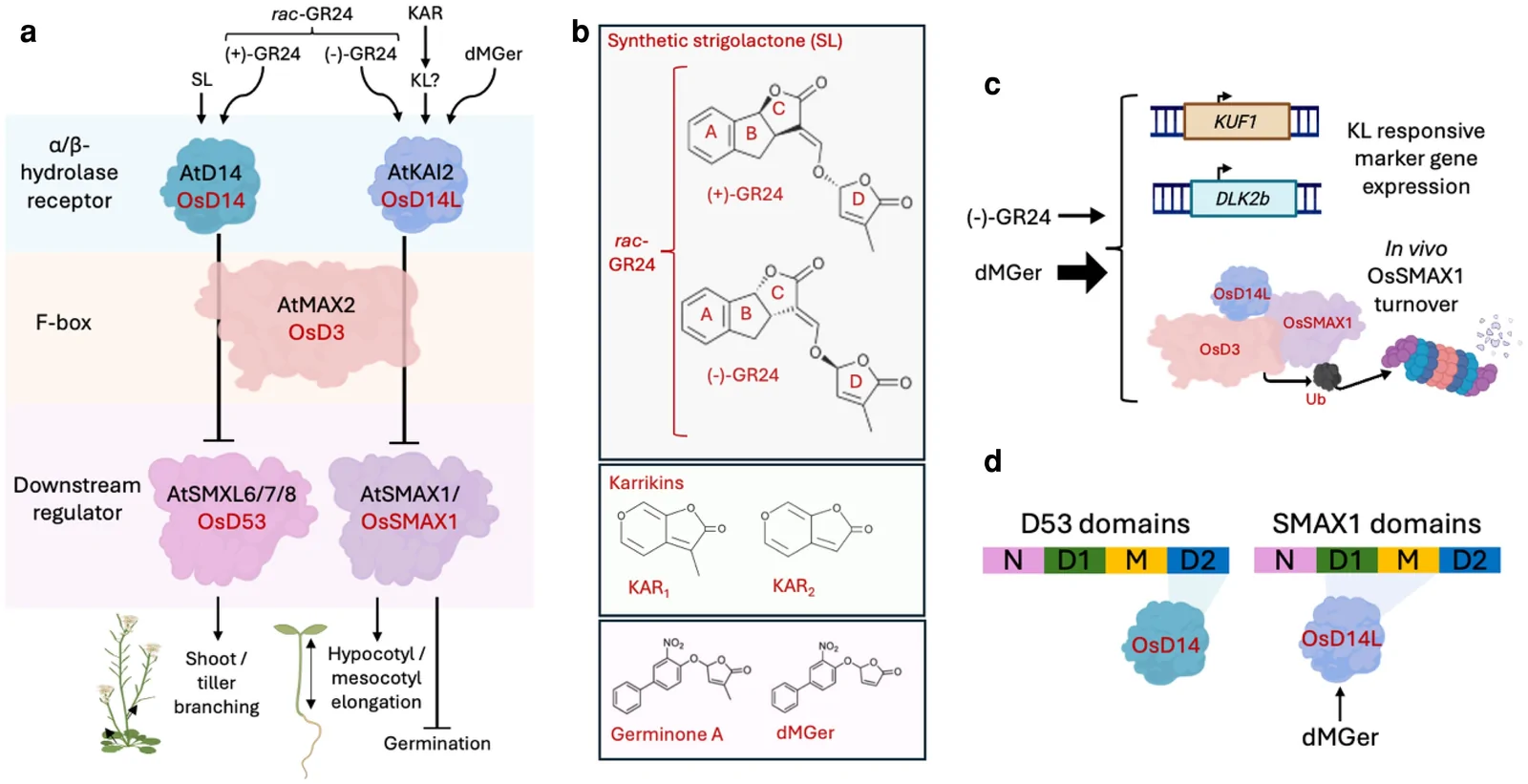
A) Strigolactone (SL) and karrikin (KAR) signaling pathway components. Both pathways are characterized by α/β-hydrolase receptors that cooperate with an F-box–containing protein (MAX2/D3) to ubiquitinate downstream regulators for proteasomal turnover to control aspects of plant development. Gene names are given for Arabidopsis (upper, black text) and rice (lower, red text). Ligands for each receptor are shown at the top. B) The commonly used synthetic strigolactone (SL), *rac*-GR24, is a racemic mixture of naturally occurring (+)-GR24 and the nonnatural enantiomer, (−)-GR24. (+)-GR24 activates signaling through D14, and (−)-GR24 can activate KAI2. KARs are smoke-derived compounds thought to be metabolized into an unknown endogenous KAI2 ligand (KL). Desmethyl germinone A (dMGer) is a recently derived compound from germinone A that was proposed to more specifically activate the KAI2 pathway in Arabidopsis. C) Summary of *in vivo* evaluation of KL-responsive gene reporters and SMAX1 protein stability in response to (−)-GR24 and dMGer. D) Proposed model of SL or KL α/β-hydrolase receptor binding to distinct domains of D53 or SMAX1. Cartoons created by BioRender.

Intriguingly, KARs also induce developmental responses in non-fire-following plants, such as *Arabidopsis thaliana* (Arabidopsis) and rice, including the promotion of germination and inhibition of hypocotyl/mesocotyl elongation (Conn and Nelson 2016; Martinez et al 2022). However, KARs are not naturally found in living plants, and *in vitro*, they do not alter KAI2 thermostability, a measure of ligand binding, suggesting KARs do not directly bind KAI2 (Conn and Nelson 2016). Together, these observations suggest KAI2 is an “orphan receptor” that perceives an unknown endogenous ligand that is neither SL nor KAR. Instead, KARs are predicted to be metabolized *in planta* to the bioactive KAI2 ligand, called KL (Conn and Nelson 2016). This presents an unusual case whereby the KL signaling pathway has been elucidated in the absence of the endogenous ligand (Fig. 1a).

Work has aimed to identify ligands that more closely mimic KL and act as better KAI2 agonists. Since the SL receptor D14 shares structural similarities with KAI2, it is reasonable that certain structural features of SLs might inform on the nature of KL. Indeed, a commonly used synthetic SL, GR24, is synthesized as a racemic mixture of two stereoisomers. And while the naturally occurring (+)-GR24 is perceived by D14, the nonnaturally occurring stereoisomer, (−)-GR24, induces KAI2 signaling (Scaffidi et al 2014). Yet high concentrations of (−)-GR24 are required to induce thermostability shifts to KAI2, suggesting it may have low specificity or affinity for KAI2 and is therefore not an ideal agonist (Fukui et al 2019). A recent screen for compounds that more strongly and selectively activated the KAI2 pathway identified germinone A (Fukui et al 2019). Subsequently, a desmethyl derivative of germinone A (dMGer; Fig. 1b) was made that induced *in vivo* KAI2 responses and greater thermostability shifts to KAI2 but not D14. Moreover, dMGer induced a KAI2–SMAX1 interaction in yeast and germination *in planta* (Okabe et al 2023). Overall, dMGer was proposed as a specific and potent KAI2 agonist compared with either germinone A or (−)-GR24, providing a useful tool to dissect KAI2 signaling in the absence of KL. However, its efficacy has so far only been assessed in Arabidopsis.

Beyond Arabidopsis, there is significant interest in understanding the KAI2/D14L signaling pathway in rice since, unlike Arabidopsis, rice forms arbuscular mycorrhizal (AM) symbioses with soil fungi to facilitate nutrient acquisition, and the formation of this symbiosis depends on the D14L-D3-SMAX1 pathway (Choi et al 2020). Recently in *Plant Physiology*, Tanaka et al (2026) used the KL analogs dMGer and (−)-GR24 to study the *in vitro* physical interactions among D14L, D3, and SMAX1 from rice. Since rice had not previously been shown to respond to dMGer, the authors first assessed its effects on dark-grown rice mesocotyl elongation. As expected, based on analogy with Arabidopsis hypocotyls, rice mesocotyls were shorter upon dMGer treatment, and this was *D14L*-dependent, placing dMGer as a KL analog in rice.

To compare the efficacy of dMGer with the more widely studied compound, (−)-GR24, Tanaka et al (2026) investigated two additional KL signaling outputs (Fig. 1c). First, they profiled gene expression changes for homologs of two KL-responsive reporters from Arabidopsis: *D14-LIKE2b* (*DLK2b*) and *KARRIKIN-UP-REGULATED F-BOX1* (*KUF1*). For both reporter genes, dMGer resulted in stronger induction than (−)-GR24. Additionally, (−)-GR24 but not dMGer was able to induce expression of *DLK2* and *KUF1* in a *d14l* loss-of-function background, suggesting that while (−)-GR24 may have other targets in rice, dMGer is specific to D14L. Second, they assessed *in vivo* stability of SMAX1, the key downstream repressor protein in the KL pathway (Fig. 1a). While (−)-GR24 and dMGer both reduced SMAX1 levels in a *D14L*-dependent fashion, SMAX1 degradation was promoted at an order of magnitude lower concentration by dMGer compared with (−)-GR24. Thus, dMGer was concluded to be a stronger and more specific KL analog in rice than the widely used (−)-GR24.

The authors next made use of a pro-fluorescent KL analog, desmethyl Yoshimulactone Green (dYLG), that releases a fluorescent product following hydrolysis. A methylated version of this compound (YLG) was originally developed to study SL receptor hydrolysis in parasitic plants, but chemical variations have since found wide utility in studying hydrolysis kinetics *in vitro* and *in vivo* for a range of α/β-hydrolase proteins of the D14/KAI2/D14L receptor family (Tsuchiya et al 2015). While dMGer could competitively inhibit dYLG hydrolysis at 1 µM, (−)-GR24 could not do so even at 10 µM. Moreover, only dMGer induced thermostability shifts in differential scanning fluorometry assays. Together, this demonstrated that dMGer, but not (−)-GR24, likely binds to the D14L ligand-binding pocket *in vitro*. Yet (−)-GR24 does induce KL phenotypes in rice, indicating that, as suggested for KAR (Conn and Nelson 2016), (−)-GR24 may be metabolized to a suitable KL analog in rice prior to perception by D14L or another similar receptor.

The next question was whether dMGer binding to D14L affected protein–protein interactions with its signaling partners, D3 and SMAX1. To answer this, Tanaka et al (2026) examined these interactions using *in vitro* pull-down assays. Without a ligand, D14L weakly interacted with D3 and more strongly with SMAX1. Both interactions were enhanced by dMGer but not (−)-GR24. Together, this hinted that ligand binding may not be required to assemble the signaling components together but rather triggers a conformational change within the preformed complex that leads to downstream signaling.

Since the SL and KL signaling pathways share a common F-box protein, D3 (Figure 1a), a key question for the field is: what confers specificity to the D14 or D14L pathway to direct D3 to distinct downstream targets, either D53 or SMAX1, respectively? Proteins of the SMAX1/D53 family show weak homology to HEAT SHOCK PROTEIN101 (AtHSP101) and are characterized by an N-terminal double Clp-N motif, a putative ATPase domain 1 (D1), a middle region (M), and a second C-terminal putative ATPase domain (D2) (Khosla et al 2020). Previous work found D14 interacted solely with the D53-D2 domain and was therefore proposed to be important for D14-dependent D53 turnover via D3 (Shabek et al 2018). In this study, Tanaka et al (2026) found that D14L only weakly interacted with the D53-D2 domain *in vitro*, and this interaction appeared not to differ when tested with the paralogous domain from SMAX1 (SMAX1-D2). However, when testing another SMAX1 domain, SMAX1-D1M, the authors demonstrate a strong interaction with D14L that was further induced by dMGer (Figure 1d). This aligns with prior work showing yeast-2-hybrid–based interactions between KAI2 and SMAX1-D1M from Arabidopsis (Khosla et al 2020). Thus, although D14 and D14L are closely related paralogs, they appear to preferentially interact with distinct domains on their cognate downstream repressor proteins (Figure 1d), potentially providing a mechanism for targeted ubiquitination. Going forward, *in vivo* functional data would help further strengthen this *in vitro* biochemical model.

Overall, Tanaka et al have demonstrated that dMGer activates signaling through D14L in rice, permitting biochemical studies of signaling component interactions in the absence of KL and demonstrating distinct binding modes of D14 and D14L to their downstream repressors. That dMGer selectively activates D14L rather than D14 signaling opens chemical routes to manipulate specific plant phenotypes in this crop plant, such as germination and AM symbiosis, without interfering with phenotypes regulated by SLs, such as tillering. Furthermore, dMGer may prove useful in chemical genetic screens to identify KL or genes required for KL biosynthesis.

**Recent related articles in *Plant Physiology*:**

- Martinez et al (2022) investigated the basis for high sensitivity of lettuce germination in response to KAR_1_. Cross-specific complementation experiments in Arabidopsis of the 2 lettuce KAI2 paralogs suggested LsKAI2b is responsible for responding to KAR_1_. The authors go on to conduct a detailed analysis of receptor pocket residues that are likely important for ligand specificity.

## Data Availability

No new data were generated or analysed in support of this research.
